# Small-Molecule Synthetic Compound Norcantharidin Reverses Multi-Drug Resistance by Regulating Sonic Hedgehog Signaling in Human Breast Cancer Cells

**DOI:** 10.1371/journal.pone.0037006

**Published:** 2012-05-15

**Authors:** Yu-Jen Chen, Cheng-Deng Kuo, Szu-Han Chen, Wei-Jen Chen, Wen-Chien Huang, K. S. Clifford Chao, Hui-Fen Liao

**Affiliations:** 1 Department of Radiation Oncology, Mackay Memorial Hospital, Taipei, Taiwan; 2 Institute of Traditional Medicine, National Yang Ming University, Taipei, Taiwan; 3 Department of Medical Research and Education, Taipei Veterans General Hospital, Taipei, Taiwan; 4 Department of Biochemical Science and Technology, National Chiayi University, Chiayi, Taiwan; 5 Division of Thoracic Surgery, Department of Surgery, Mackay Memorial Hospital, Taipei, Taiwan; 6 Department of Radiation Oncology, Columbia University, New York, New York, United States of America; National Cancer Center, Japan

## Abstract

Multi-drug resistance (MDR), an unfavorable factor compromising treatment efficacy of anticancer drugs, involves upregulated ATP binding cassette (ABC) transporters and activated Sonic hedgehog (Shh) signaling. By preparing human breast cancer MCF-7 cells resistant to doxorubicin (DOX), we examined the effect and mechanism of norcantharidin (NCTD), a small-molecule synthetic compound, on reversing multidrug resistance. The DOX-prepared MCF-7R cells also possessed resistance to vinorelbine, characteristic of MDR. At suboptimal concentration, NCTD significantly inhibited the viability of DOX-sensitive (MCF-7S) and DOX-resistant (MCF-7R) cells and reversed the resistance to DOX and vinorelbine. NCTD increased the intracellular accumulation of DOX in MCF-7R cells and suppressed the upregulated the mdr-1 mRNA, P-gp and BCRP protein expression, but not the MRP-1. The role of P-gp was strengthened by partial reversal of the DOX and vinorelbine resistance by cyclosporine A. NCTD treatment suppressed the upregulation of Shh expression and nuclear translocation of Gli-1, a hallmark of Shh signaling activation in the resistant clone. Furthermore, the Shh ligand upregulated the expression of P-gp and attenuated the growth inhibitory effect of NCTD. The knockdown of mdr-1 mRNA had not altered the expression of Shh and Smoothened in both MCF-7S and MCF-7R cells. This indicates that the role of Shh signaling in MDR might be upstream to mdr-1/P-gp, and similar effect was shown in breast cancer MDA-MB-231 and BT-474 cells. This study demonstrated that NCTD may overcome multidrug resistance through inhibiting Shh signaling and expression of its downstream mdr-1/P-gp expression in human breast cancer cells.

## Introduction

Breast cancer is a common malignant tumor in woman. Surgical excision, chemotherapy, irradiation, and hormone therapy are the major therapeutic options. Recently, targeted therapy, such as Trastuzumab and lapatinib to specifically inhibit the activity of receptors HER2/neu and epidermal-derived growth factor receptor (EGFR1), respectively, has already become clinically established as an effective and less toxic strategy for the treatment of breast cancer [1], [2]. However, more and more research is indicating that tumor cell resistance to chemotherapeutic drugs signals failure for the cancer treatment [3].

Multidrug resistance (MDR) is an unfavorable factor causing the failure of treatments against cancer cells [4]. It occurs when cancer cells acquire simultaneous resistance to various kinds of chemotherapeutic agents with no structural or functional similarities [5]. One of the mechanisms involved in MDR that reduces intracellular drug accumulation is the active efflux of chemotherapeutic agents through membrane drug transporters, especially the ATP-binding cassette (ABC) proteins, including p-glycoprotein (P-gp, MDR1, ABCB1), the multidrug resistance protein 1 (MRP1), and the breast cancer resistance protein (BCRP, ABCG2) [6].

Sonic hedgehog (Shh) is a secretory signaling protein involving embryogenesis that was first discovered in *Drosophila* [7]. Upon binding the Shh ligand to its transmembranous receptor PTCH1, the PTCH1-mediated inactivation of Smoothened (Smo) is reversed to activate the Shh signaling pathway, resulting in translocation of the cytoplasmic transcription factors Gli family into the nucleus to modulate the expression of target genes controlling the cell cycle, cell adhesion, signal transduction, vascularization, and apoptosis [8]. We previously showed that Sonic hedgehog (Shh) signaling pathway activation is associated with resistance to chemoradiation in esophageal adenocarcinoma [9] and resistance to multiple structurally unrelated chemotherapeutic agents through regulating MDR1 and BCRP expression [10]. This link of MDR and Shh signaling may provide a novel target for the development of anticancer drugs reversing MDR. Moreover, a recent article reported that the expression between nuclear FOXC2 and Gli-1 has a significant correlation in estrogen receptor (ER)-negative breast cancers, resulting in a poor rate of disease-free survival [11]. By mutation analysis of 36 novel candidate cancer genes in 96 human breast cancers, demonstrating that potential impact on protein function in the genes related to Notch, Hedgehog, NF-κB, and PIK3CA pathways [12]. However, there was no research mentioned about the correlation between Shh and MDR in breast cancers. Thus, clarification for correlation between Shh signaling and MDR is crucial for developing a new possible strategy for optimizing clinical treatment of breast cancers.

Norcantharidin (NCTD) is a small-molecule synthetic demethylated analog of the naturally occurring cantharidin isolated from blister beetles (*Mylabris phalerata* Pall.). The anticancer effects of NCTD have been investigated and reported against a diversity of malignancies by inducing cell anoikis and apoptosis [13], inhibiting invasion and angiogenesis [14], and suppressing metastasis [15]. Unlike the conventional chemotherapeutics, NCTD is preferentially toxic to cancer cells rather than normal cells [16], making this small molecule promising in cancer treatment. Further, our preliminary work indicates that NCTD may possess pharmacological activity for reversing drug resistance in cancer cells.

To clarify the effect of NCTD on drug resistance in breast cancer, we developed doxorubicin (DOX)-sensitive and DOX-resistant MCF-7 cells [17]. The possible mechanisms of action, including cell viability, drug efflux, MDR molecule expression, Shh signaling regulation, the correlation between MDR and Shh, and the above effects in the other breast cancer cell lines, were also investigated.

## Materials and Methods

### Materials and cells

The NCTD (exo-7-oxabicylo-[2.2.1] heptane-2,3-dicarboxylic anhydride) with analytical grade purity, doxorubicin (DOX), cyclosporine A (CsA), and the chemicals used in this study were purchased from Sigma (St. Louis, MO). Vinorelbine (VNR) and Sonic Hedgehog N-terminal peptide were purchased from GlaxoSmithKline (Middlesex, UK) and R&D systems (Minneapolis, MN), respectively. Human breast adenocarcinoma MCF-7 cells (estrogen receptor (ER)-positive, progesterone receptor (PR)-positive, and HER2-low), sensitive to the chemotherapeutic drug DOX (denoted as MCF-7S), were purchased from the American Type Culture Collection (Manassas, VA) and were cultured in alpha minimum essential medium (α-MEM, Invitrogen, Carlsbad, CA) supplemented with 10% heat-inactivated fetal bovine serum (FBS), 1.5 g/L NaHCO_3_, and 2 mM L-glutamine. MCF-7R cells were developed as DOX-resistant according to the method reported by Mechetner et al [17]. In brief, *in vitro* culture of MCF-7 cells was grown in the same medium containing DOX with the initial concentration of 10 nM. The survival cells with drug resistant effect were selected by passaging cells in increasing DOX concentrations till 150 nM. Cultures were maintained in a humidified atmosphere with 5% CO_2_ at 37°C. The cultured cells were subcultured twice each week, seeding at a density of about 2×10^5^ cells/mL. Additionally, human breast cancer cell lines BT-474 (HER2^+^ ER^+^ PR^+^) and MDA-MB-231 (HER2^−^ ER^−^ PR^−^) cells, kindly provided from Dr. Huang WC (Mackay Memorial Hospital, Taipei, Taiwan), were cultured in Dulbecco's modified Eagle's medium (Invitrogen, Carlsbad, CA) supplemented with 10% FBS, 1.5 g/L NaHCO_3_, and 2 mM L-glutamine.

### Viability of breast cancer cells

MCF-7S and MCF-7R cells were cultured and treated with DOX (0–300 nM), NCTD (0–50 µM), VNR (0.5 nM), Shh peptide (0.1 µg/mL), and/or CsA (4 µM) for 48 h. Cells were then collected and the cell viability (%) was determined by using the 3-(4,5-dimethylthiazol-2-yl)-2,5-diphenyltetrazolium bromide (MTT, Sigma) colorimetric assay and the trypan blue dye exclusion method. BT-474 and MDA-MB-231 cells were treated with DOX (150 nM), NCTD (10 µM), and Shh peptide (0.1 µg/mL) for 48 h and then assayed the viability by trypan blue dye exclusion method.

### DOX efflux

MCF-7S and MCF-7R cells with DOX (150 nM) and/or NCTD (10 µM) treatment were harvested for 48 h. DOX could emit red fluorescence under the excitation wave-length at 510–550 nm. For assessment of DOX accumulation, the cells were collected, washed by phosphate-buffered saline (PBS), and the phenotype of the cells was observed under a fluorescence microscope at a magnification of 400× for measuring the percentage of cells with intracellular DOX accumulation (%).

### RNA extraction and reverse transcription (RT)-PCR assay of mdr-1, Shh, and Smo expression

The total RNA was extracted from the cells using TRIzol reagent (Invitrogen) in accordance with the manufacturer's instructions. The RNA was reverse-transcribed with Superscript II Transcriptase (Invitrogen) in the presence of oligo-dT and random primers. For the polymerase chain reaction (PCR) assay, the primer sequences and PCR condition for the MDR-1, Shh, Smo, and glyceraldehyde-3-phosphate dehydrogenase (GAPDH) genes are shown in Table 1. After PCR, the products were analyzed on 1% agarose gels stained with ethidium bromide (0.01%). Relative band intensities were determined using ImageJ software (Version 1.36b, National Institute of Health, Bethesda, Maryland). Expression levels of the GAPDH gene were used for standardization. Each experiment was repeated at least three times.

**Table 1:** Primer sequences used for the RT-PCR assay.

Genes	Sequences	Annealing temp.^a^	Products (bp)
***mdr-1*** **:**		53	312
Forward	5′-AAAGCTGTCAAGGAAGCCAA-3′		
Reverse	5′-TGACTCCATCATCGAAACCA-3′		
***Shh*** **:**		33.8	477
Forward	5′-CGCACGGGGACAGCTCGGAAGT-3′		
Reverse	5′-CTGCGCGGCCCTCGTAGTGC-3′		
***Smo*** **:**		50.7	322
Forward	5′-TTACCTTCAGCTGCCACTTCTACG-3′		
Reverse	5′-GCCTTGGCAATCATCTTGCTCTTC-3′		
***GAPDH*** **:**		55	574
Forward	5′-CCACCCATGGCAAATTCCATGGCT-3′		
Reverse	5′-TCTAGACGGCAGGTCAGGTCCACC-3′		

a The PCR thermal cycle profile consisted of 1 cycle of denaturation for 5 min at 95°C; 35 cycles of denaturation for 30 sec at 95°C, annealing of primers for 45 sec at different temperatures as shown in this Table, and extension for 1 min at 72°C; and 1 cycle of a final extension step at 72°C for 10 min.

### Western blot analysis of P-gp, MRP1, BCRP, and Shh expression

The protein was extracted from the cells with lysis buffer (8 M urea and 4% 3-[(3-cholamidopropyl)dimethylammonio]-1-propanesulfonate detergent, Sigma) at 4°C. Then, the supernatants containing total protein were separated by centrifugation at 10,000 g for 30 min. Protein samples (50 µg) were mixed with 2× concentrated electrophoresis sample buffer (1 M Tris, pH 6.8, 5% SDS, 40% glycerol, 0.005% bromophenol blue, and 8% β-mercaptoethanol), separated on a 10% SDS-polyacrylamide gel, and transferred to polyvinylidene fluoride (PVDF) membranes. After blocking, the blots were respectively incubated with primary antibody directed against P-gp (1∶200; Santa Cruz Biotechnology, Heidelberg, Germany), MRP-1 (1∶200; Santa Cruz), BCRP (1∶1000; Santa Cruz), Shh (1∶500; Santa Cruz), or GAPDH (1∶1000, Santa Cruz) overnight at 4°C. The results were detected using horseradish peroxidase (HRP)-conjugated anti-mouse IgG (1∶10,000 for 1 h at room temperature) followed by enhanced chemiluminescence using ECL reagents and analyzed using a chemiluminescence imaging system (Perkin Elmer, Waltham, Massachusetts). The protein bands were then analyzed using the ImageJ software. The mean values were normalized to the internal GAPDH control and were calculated from at least three independent experiments.

### Immunofluorescence staining of Gli-1 distribution

The treated cells were collected and reacted with anti-Gli-1 primary antibody (1∶1000, Santa Cruz) and immunofluorescence PE-conjugated anti-IgG-TR antibody (1∶500, Santa Cruz) in order to determine the distribution of Gli-1 expression in cells. Hoechst 33342 fluorescence dye was also used to stain the location of the nucleus. The cells were then photographed under a fluorescence microscope at a magnification of 400×.

### Correlation between P-gp and Shh signaling by mdr-1 siRNA assay

To assess the role of P-gp on Shh signaling regulation, we used mdr-1 siRNA (HSS182278) and negative control siRNA (Invitrogen). In brief, the cells were transfected with 20 µM of siRNA using lipofectamine™ RNAiMAX reagent (Invitrogen). After 48 h, the cells were collected and the mRNA levels of mdr-1, Shh, and Smo were analyzed by reverse transcription polymerase chain reaction (RT-PCR) assay. Relative band intensities were determined using ImageJ software. The mean values were normalized to the internal GAPDH control and were calculated from at least three independent experiments.

### Data analysis

Results were expressed as the mean ± standard error (SE) from at least three experiments. Statistical comparisons were performed using Student's *t*-test or analysis of variance as appropriate. Differences were considered significant at a *P* of less than 0.05. All statistical analyses were carried out using GraphPad Prism 4 (San Diego, CA, USA) and SigmaPlot software (Version 9.0, Systat Software Inc., San Jose, CA).

## Results

### Effect of NCTD on breast cancer MCF-7 cells possessing MDR

**Figure 1:**
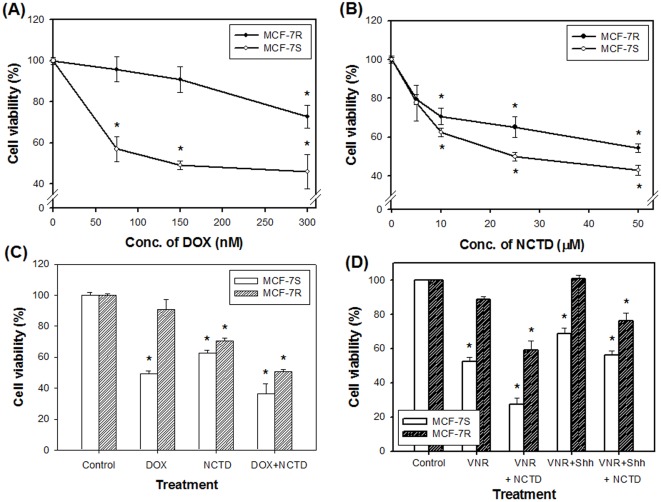
Viability of the MCF-7R and MCF-7S cells. Cells were treated with (**A**) DOX (0–300 nM), (**B**) NCTD (0–50 µM), (**C**) a combination of DOX (150 nM) and/or NCTD (10 µM), and (**D**) a combination of VNR (0.5 nM), Shh peptide (0.1 µg/mL) and/or NCTD (10 µM). After 48 h, the cell viability was assayed by the MTT method. The results were expressed as the mean ± SD of at least three independent experiments. *Significant difference compared with the untreated control group.

In a pair of MCF-7 cell subclones, with doxorubicin-sensitive (MCF-7S) and doxorubicin-resistant (MCF-7R) characteristics established previously (Figure 1A) by MTT colorimetric assay, NCTD inhibited the viability of both cell lines in a concentration-dependent manner (Figure 1B). At suboptimal concentration (10 µM), NCTD markedly promoted the growth inhibitory effect of DOX (Figure 1C). The DOX-prepared resistant cells were also resistant to vinorelbine (VNR), indicating acquirement of MDR (Figure 1D). We found that NCTD was effective at enhancing the growth inhibition of VNR against MCF-7R cells (Figure 1D). Given that resistance to agents with no structural or mechanistic similarities may be mediated by modulating the drug efflux transporters, we next examined the effect of the known drug pump modulator Shh ligand on drug activity. Intriguingly, Shh N-terminal peptide subverted the growth inhibitory activity of VNR and partially blocked the enhancing effect of NCTD (Figure 1D). Moreover, cell viability measured by the Trypan blue dye exclusion method had a similar result as when tested by the MTT method (data not shown).

### Effect of NCTD on intracellular accumulation of DOX and expression of drug efflux transport proteins

**Figure 2:**
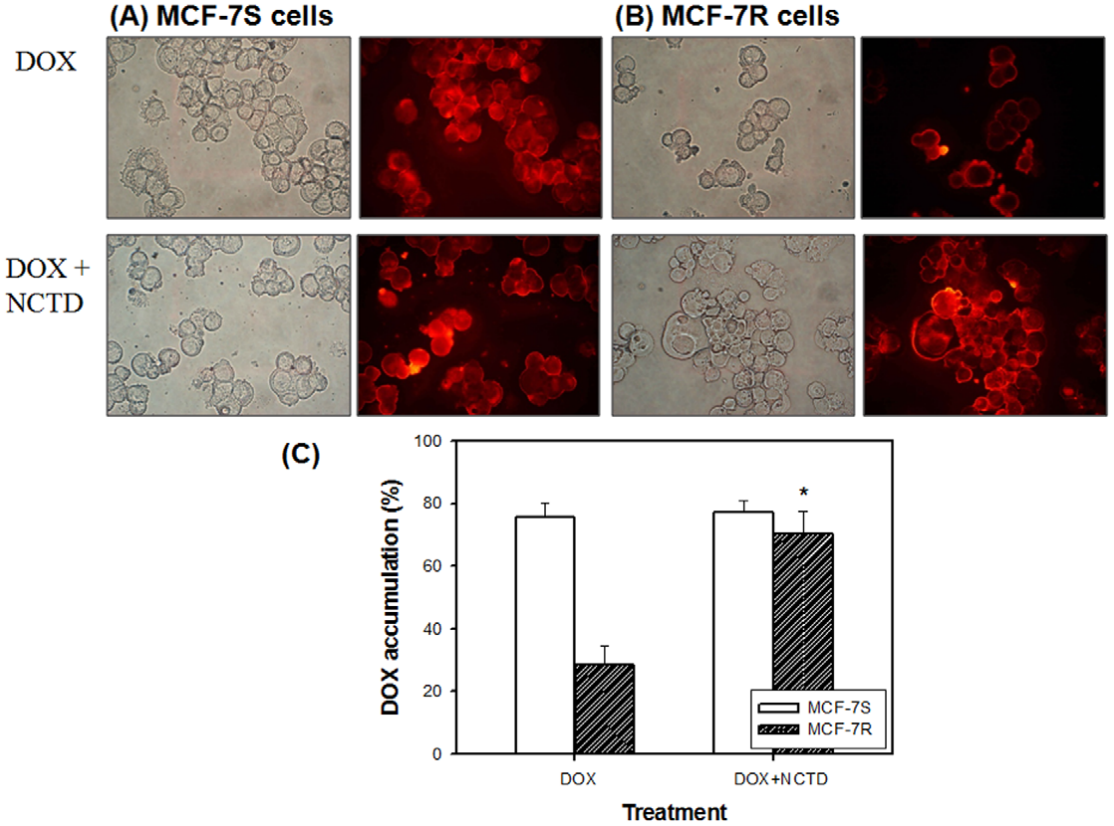
Effect of NCTD on the distribution of DOX in cells. (**A**) MCF-7S and (**B**) MCF-7R cells were treated with DOX (150 nM) and/or NCTD (10 µM) for 48 h. Then, the cells were washed by PBS, observed under a fluorescence microscope (400×), and (**C**) The percentage of cells with intracellular DOX accumulation was measured. *Significant difference between DOX alone and DOX+NCTD in the same cell type.

**Figure 3:**
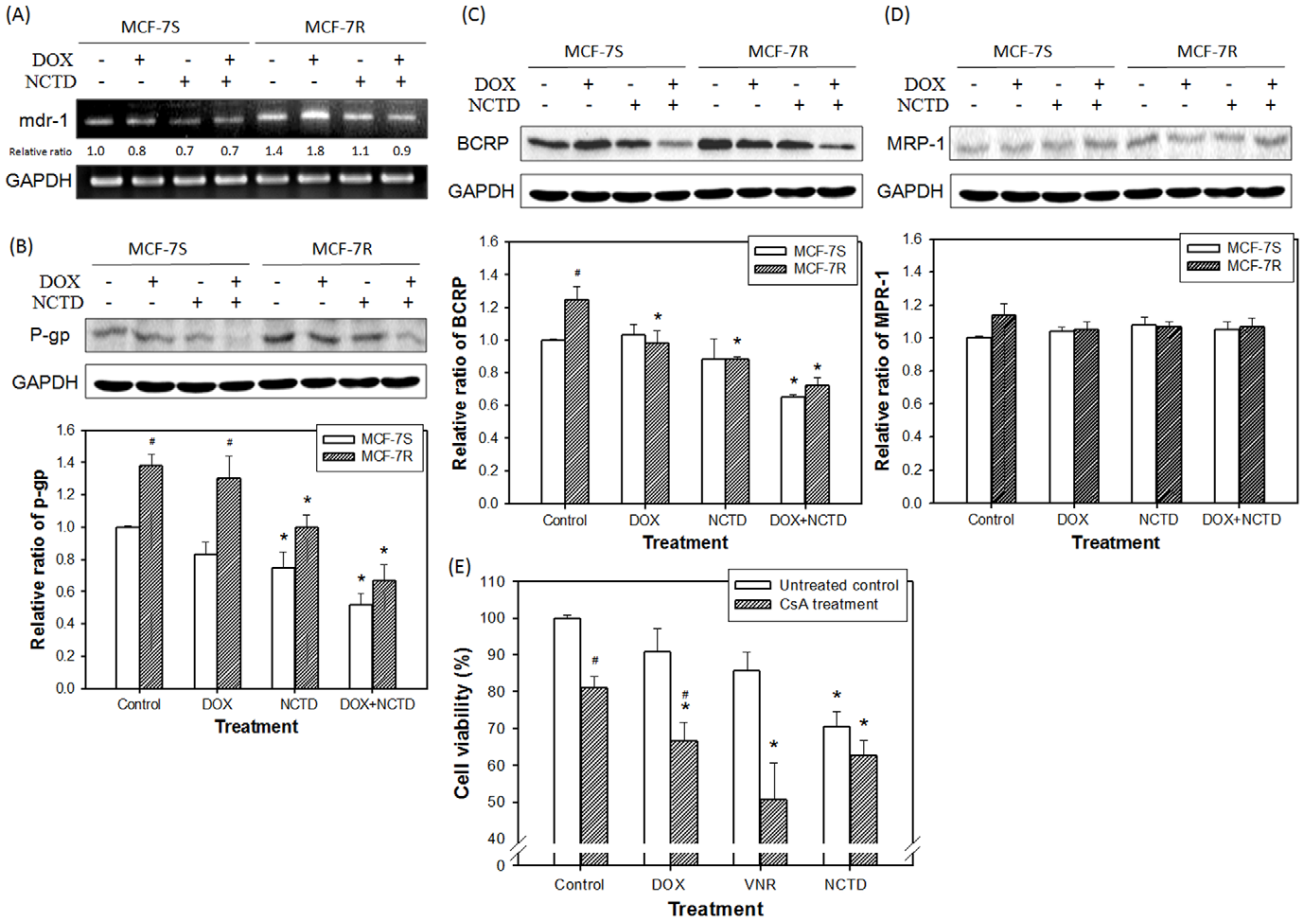
Expression of mdr-1/P-gp, BCRP, and MPR-1 in MCF-7S and MCF-7R cells. (**A**) RT-PCR assay of mdr-1 mRNA level in DOX and/or NCTD-treated cells. Western blotting of (**B**) P-gp, (**C**) BCRP, and (**D**) MPR-1 expression in DOX and/or NCTD-treated cells. (**E**) Viability of DOX, VNR, and NCTD in MCF-7R cells with or without CsA (4 µM) treatment. Cells were cultured with a DOX (150 nM) and/or NCTD (10 µM) treatment for 48 h. Then, the cells were washed by PBS and their total RNA and proteins for RT-PCT and Western blot analysis, respectively, were extracted. To clarify the role of MDR-related gene and protein expression, MCF-7R cells were treated with or without CsA, and then assayed for cell viability. GAPDH was used as the internal control. The relative expression of each band was assayed and the data were expressed as the mean ± SD of at least three independent experiments. *Significant difference as compared to the untreated control. ^#^Significant difference between MCF-7S and MCF-7R (B–D) and the CsA treatment and non-treatment (E) at the same treatment of NCTD.

By using the fluorescent property of DOX, we noted that the intracellular accumulation of DOX was significantly reduced in the MCF-7R cells as compared to the MCF-7S cells (Figure 2). The reduction of DOX accumulation was reversed by the NCTD treatment in the MCF-7R cells (Figure 2B and 2C). This data supported the effect of NCTD on drug efflux transport proteins. To further elucidate the involved proteins, we assessed their expression at the mRNA and protein levels. As demonstrated in Figure 3A and 3B, both the expression of mdr-1 mRNA and its transcribed P-gp protein were upregulated in the MCF-7R cells. This upregulation was markedly inhibited by the NCTD treatment. Moreover, the expression of the other ABC proteins (BRCP, but not MRP-1) had a similar alteration profile as P-gp (Figures 3C and 3D). CsA, a pharmacological inhibitor of P-gp function, partially reversed the DOX and VNR but not the NCTD resistance in the MCF-7R cells (Figure 3E), supporting the role of P-gp in this MDR model and indicating the pump sparing effect on NCTD.

### Effect of NCTD on Shh signaling and link of P-gp expression

**Figure 4:**
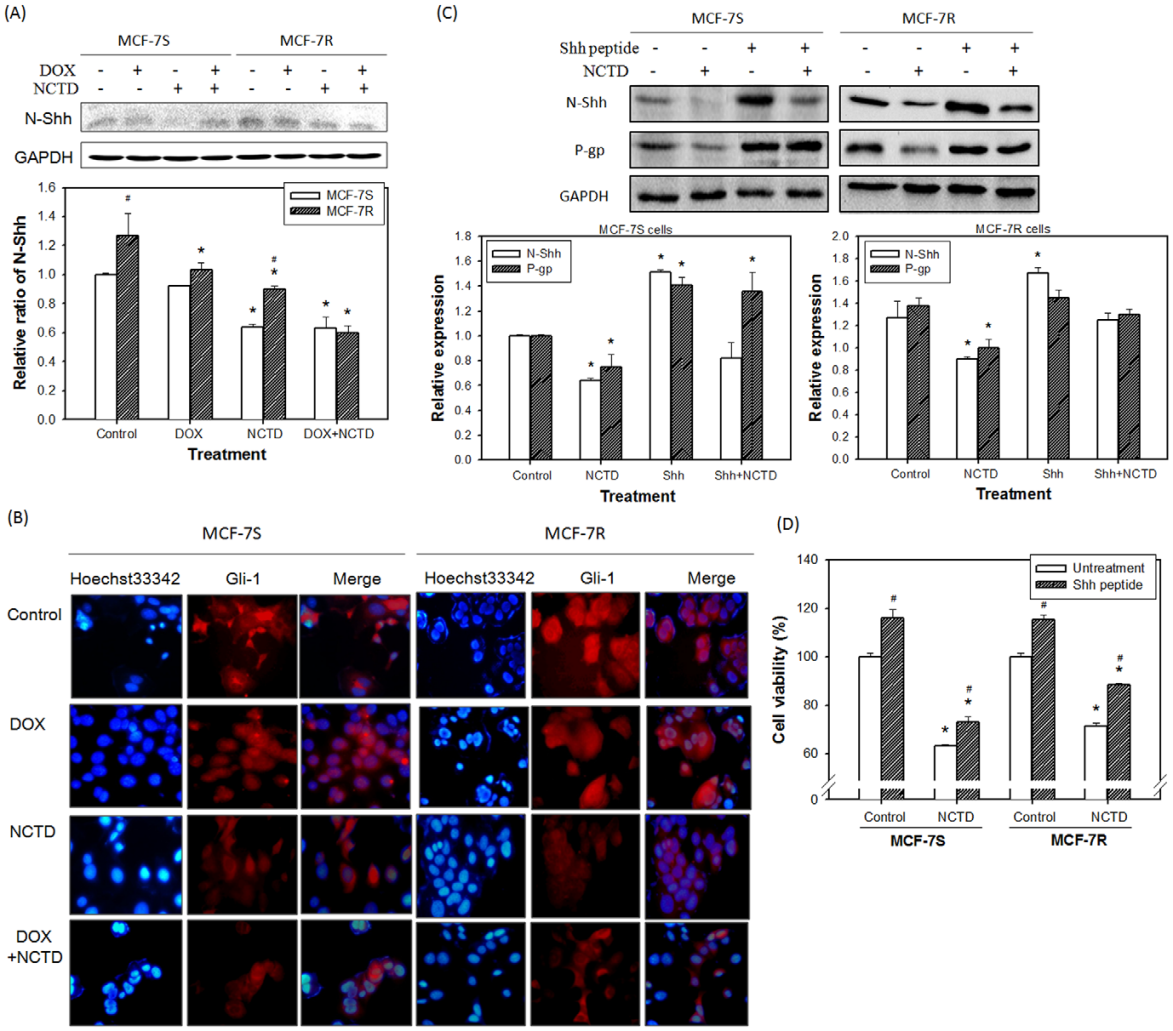
Effect of Shh signaling in cells with and without DOX and/or NCTD treatment. (**A**) Expression of N-Shh by Western blot assay. (**B**) Gli-1 translocation by immunofluorescence assay. (**C**) Western blot analysis of N-Shh and P-gp expression in cells with and without Shh peptide and/or NCTD treatment. (**D**) Cell viability assay of cells with and without Shh peptide and/or NCTD treatment. MCF-7S and MCF-7R cells were cultured with DOX (150 nM) and/or NCTD (10 µM) treatment for 48 h. Then, the cells were washed by PBS and their total proteins extracted for Western blot analysis. For the Gli-1 translocation assay, the cells were stained with Hoechst33342 and fluorescence-conjugate anti-Gli-1 antibody, and then observed under a fluorescence microscope. To clarify the role of Shh on multidrug resistance, the Shh peptide and/or NCTD were treated in the cells, and the N-Shh and P-gp expression and cell viability were assayed by Western blotting and the MTT method, respectively. GAPDH was used as the internal control and the results were confirmed by at least three independent experiments. *Significant difference as compared to the untreated control. ^#^Significant difference between MCF-7S and MCF-7R (A) and the Shh peptide treatment and non-treatment (D) at the same treatment of DOX and/or NCTD.

**Figure 5:**
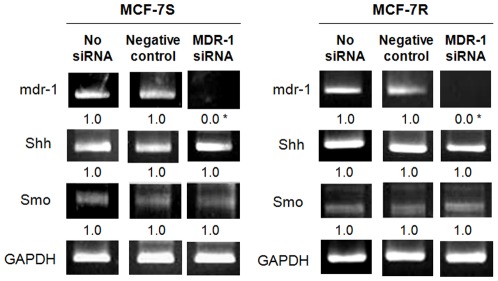
Expression of mdr-1, Shh, and Smo in cells with and without mdr-1 siRNA treatment. To clarify the role of P-gp on the regulation of Shh signaling molecules, mdr-1 siRNA was used to knock down the P-gp expression in both the MCF-7S and MCF-7R cell lines. Expression of mdr-1, Shh, and Smo mRNA levels was analyzed by RT-PCR assay. GAPDH was used as the internal control and the results were confirmed by at least three independent experiments. *Significant difference as compared to the untreated control.

As shown in Figure 4A, the expression of Shh was upregulated in the MCF-7R cells as compared to the MCF-7S clone. By the NCTD treatment, the Shh expression was greatly suppressed in both cell lines (Figure 4A). The nuclear translocation of Gli-1, a hallmark of Shh signaling activation, exhibited a similar pattern by NCTD treatment (Figure 4B). The exogenous addition of the Shh ligand increased the basal expression of Shh and P-gp in the MCF-7S cells (Figure 4C), suggesting a possible regulatory role of Shh upstream P-gp. This increase in MCF-7R cells was not evident, which may be due to high basal expression. The addition of the Shh ligand, validated by the enhanced expression of Shh (Figure 4C), moderately reduced the growth inhibitory effect of NCTD in both the MCF-7S and MCF-7R cells (Figure 4D). To further exclude the possibility that P-gp acts upstream of Shh, we knocked down the mdr-1 gene and found no significant changes in the expression of Shh and Smo in both the MCF-7S and MCF-7R cells (Figure 5). Taken together, establishment of multidrug resistance in the MCF-7 cells may result in the upregulation of Shh signaling and subsequent upregulation of P-gp. NCTD treatment could reverse the multidrug resistance through suppression of these unfavorable molecular events.

### Effect in other breast cancer cells

**Figure 6:**
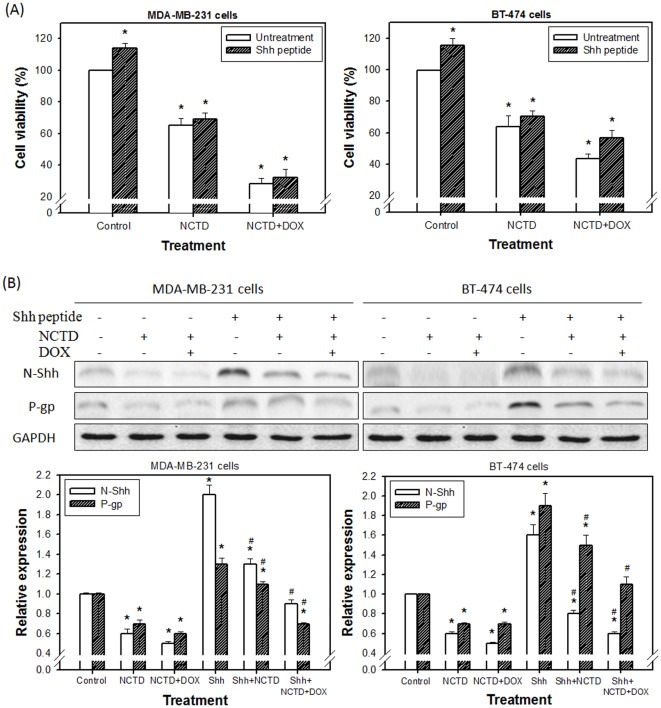
Effect of Shh signaling in human breast cancer MDA-MB-231 and BT-474 cells. (**A**) Cell viability assay. (**B**) Expression of N-Shh and P-gp by Western blotting. Cells were cultured with or without Shh peptide (0.1 µg/mL), NCTD (10 µM) or NCTD plus DOX (150 nM) treatment for 48 h. The treated cells were collected, assayed the viability by using trypan blue dye exclusion test, and then their total proteins were extracted for Western blot analysis. Expression of N-Shh and P-gp levels were normalized to GAPDH and the results were confirmed by at least three independent experiments. *Significant difference as compared to the untreated control. ^#^Significant difference as compared to the Shh alone group.

As shown in Figure 6A, cell viability were increased with Shh peptide treatment in MDA-MB-231and BT-474 cells. NCTD inhibited the cell growth and had synergistic effect with DOX to eliminate the cell viability that increased by Shh peptide. In Figure 6B, the expression of N-Shh and P-gp were also upregulated in Shh-treated MDA-MB-231and BT-474 cells, while treatment of NCTD and NCTD plus DOX effectively decreased the levels of N-Shh and P-gp. Moreover, BT-474 cells, HER2/neu, ER and PR-triple positive cells, were less sensitive to NCTD plus DOX-mediated cytotoxity and expressed higher P-gp level when the cells treated with Shh peptide.

## Discussion

The unique characteristics of NCTD include small-molecule, simple synthesis procedures [18], a leukocytosis effect instead of myelosuppression [19], and validated safety in vivo [20]. Growing evidence shows NCTD may have pharmaceutical potential to be developed as a novel category of anticancer drugs. NCTD is known as a potent anticancer drug against various types of cancer cells [13], [16]. In our previous study, we demonstrated that NCTD inhibited pulmonary metastasis of colorectal cancer CT26 cells *in vivo* and cell invasion ability *in vitro*, accompanied by the downregulation of MMP-9 [20]. Furthermore, NCTD downregulated MMP-9 promoter activity through the inhibition of Sp-1 transactivation in colorectal cancer cells [15]. In clinical practice, cancer cells' resistance to chemotherapeutic drugs usually limits the efficacy of treatment outcome. On the basis of direct cytotoxicity, we further extended the study to investigate the effect of NCTD on multidrug resistance in cancer cells.

There are two general categories related to drug resistance: acquired and intrinsic. Both the intrinsic and acquired resistance of human tumors to chemotherapy is a multifactorial event, often leading to a lack of response to several unrelated drugs (multidrug resistance or MDR). Intrinsic resistance reflects the inability of a drug to target a particular organism, such as outer membrane impermeability and efflux pumps. Decreased intracellular drug accumulation due to reduced permeability of the plasma membrane, found in the MOS/IR1 cells, is one possible mechanism and may explain the intrinsic resistance to multidrug chemotherapy for the treatment of osteosarcoma [21]. While acquired resistance is the result of mutational resistance arising from gene mutation, transmissible resistance, multidrug resistance (MDR), *etc* [21]. Mechanisms involved in MDR include overexpression of multispecific ATP-dependent drug efflux pumps, such as P-gp (MDR1, ABCB1), MRP1, and BCRP (ABCG2), that reduce the concentration of the drug available for cancer cells [6]. In HepG2 cells, an article reported that cantharidin reversed multidrug resistance via downregulation of P-gp expression [22]. This study demonstrated that NCTD increased the intracellular accumulation of DOX in the MCF-7R cells and suppressed the upregulated mdr-1 mRNA, P-gp and BCRP protein expressions, but not the MRP-1. Moreover, it concludes that NCTD may overcome multidrug resistance via a novel drug resistance pathway, sonic hedgehog (Shh) signaling.

The Shh signaling pathway has been proven to be related to resistance to chemotherapy and chemoradiation in cancer cells [23]. We found that the chemotherapy resistance in MCF-7 breast cancer cells was independent of the mechanism of drugs, indicating a correlation to drug efflux activity. This generous multidrug resistance may compromise the clinical efficacy of chemotherapeutics against cancer cells. We further demonstrated that Shh might be the upstream modulator for this generous resistance machinery, indicating that Shh signaling could be a drug development target for a broad and salvage inhibitor of drug-resistance clones in breast cancer. Additionally, HER2/neu, ER, PR, and EGFR are highly expressed in many cases of breast cancers and considered important biomarkers [24], [25]. HER2 overexpression causes high rate of cell proliferation and lymph node involvement, which is correlated with disease aggressiveness, increased rates of recurrence and poorer survival in breast cancer patients [25]. This study demonstrated that Shh-mediated tumor growth was universal in various cell lines of breast cancer, including MCF-7 (HER2^−^ ER^+^ PR^+^), MCF-7R, MDA-MB-231 (HER2^−^ ER^−^ PR^−^), and BT-474 (HER2^+^ ER^+^ PR^+^) cells. It implicates that NCTD may benefit the therapeutic efficacy against various types of breast cancer cells via inhibiting Shh signaling and MDR. In clinical practice, the survival of triple negative breast cancer patients remains unsatisfactory. An article reported that 10% to 20% of breast cancer patients express no or low levels of ER, PR and HER2, showing poor response to chemotherapeutic agents [26]. The major reason for the poorer prognosis might has been regarded no actual targets for therapeutics. Our results indicated that NCTD was effective against cell ontogeny with triple negative. Collectively, it implicates that the clinical outcome and shortage of effective treatment might be overcome by Shh inhibitors, such as NCTD and the other small molecule therapeutics according to results of this study.

Currently investigations into inhibitors for Shh signaling include Smo or Gli-1 blockers. Among these inhibitors, the Smo antagonist vismodegib (GDC-0449) has been proven effective in phase I/II clinical trials against basal cell carcinoma [27] and medulloblastoma [28]. However, a D473H mutation in Smo has been reported causing resistance to GDC-0449 in medulloblastoma [29]. Therefore, the development of various Shh signaling inhibitors to overcome resistance or as a salvage therapy should be a critical task in treating the refractory cancers. Our result indicates NCTD may be a potential candidate for both a primary and a salvage therapeutic agent against Shh-activating cancer cells.

Shh signaling has been demonstrated involving cancer angiogenesis, metastasis, invasion [30]. For example, Shh signaling could promote the metastasis of gastric cancer cells through activation of the PI3K/Akt pathway, which may lead to epithelial mesenchymal transition and MMP-9 activation [31]. NCTD has been reported to have bioactivity against these cancer cell pathways. Whether NCTD has direct chemical interaction with Shh signaling molecules, like arsenic trioxide and Gli-1 [32], or indirect regulation of the signaling pathway remains to be examined.

Collectively, this study demonstrated that NCTD was effective at inhibiting the growth of both DOX-sensitive (MCF-7S) and DOX-resistant (MCF-7R) breast cancer cell lines and reversed the cells' resistance to chemotherapeutic drugs, including DOX and vinorelbine. The upstream Shh signaling pathway was blocked in NCTD-treated MCF-7S and MCF-7R cells, and then the expression of MDR molecules was downregulated, especially mdr-1/P-gp. Moreover, the other breast cancer cell lines, including MDA-MB-231 (HER2^−^ ER^−^ PR^−^) and BT-474 (HER2^+^ ER^+^ PR^+^), also have the similar effects. In conclusion, our results suggest that the small-molecule compound NCTD may overcome multidrug resistance through inhibiting Shh signaling and its downstream mdr-1/P-gp expression in human breast cancer cells.
